# Enhanced Immunogenicity and Distinct Local Tissue Responses of Porcine Circovirus Type 2 Vaccination in Pigs Using a Needle-Free Jet Injection System

**DOI:** 10.3390/vetsci13090854

**Published:** 2026-08-23

**Authors:** Zihui Jiao, Haiyan Wang, Xiangfei Meng, Mingfa Yang, Chunyuan Dai, Zhaoxuan Zhu, Xinzi Guo, Ping Yang, Yu Lu

**Affiliations:** 1 College of Veterinary Medicine, Nanjing Agricultural University, No. 1 Weigang, Nanjing 210095, China; 2025107020@stu.njau.edu.cn (Z.J.); wang-haiyan1983@163.com (H.W.); 2020107023@stu.njau.edu.cn (X.M.); daichunyuan@stu.njau.edu.cn (C.D.); zhaoxuanzhu@njau.edu.cn (Z.Z.); 15121225@stu.njau.edu.cn (X.G.); 2 GuoTai (Taizhou) Center of Technology Innovation for Veterinary Biologicals, Taizhou 225300, China; 3 National Research Center of Engineering and Technology for Veterinary Biologicals, Institute of Veterinary Immunology and Engineering, Jiangsu Academy of Agricultural Sciences, Nanjing 210014, China; 4 State Key Laboratory for Animal Disease Control and Prevention, Harbin Veterinary Research Institute, Chinese Academy of Agricultural Sciences, Harbin 150069, China; yangmingfa@caas.cn (M.Y.)

**Keywords:** porcine circovirus type 2, needle-free injection, intradermal, vaccine delivery, immune response

## Abstract

Conventional needle-based vaccination may cause tissue injury and increase the risk of cross-contamination between animals. This study evaluated the immunological effects of delivering an inactivated porcine circovirus type 2 (PCV2) vaccine using a needle-free jet injection system compared with needle injection in pigs. Immune responses were assessed using enzyme-linked immunosorbent assay (ELISA) for PCV2-specific antibody levels on days 7, 14, and 28 post-vaccination. Cellular architecture at injection sites and differential gene expression were analyzed using immunohistochemistry and transcriptome sequencing. Results showed that pigs receiving needle-free vaccination at 0.50 mL and 0.75 mL injection volumes exhibited higher PCV2-specific antibody responses than pigs receiving 1.0 mL needle injection. Needle-free vaccination also preserved dermal collagen fiber architecture, altered antigen-presenting cell distribution, and was associated with distinct activation of immune-related pathways, particularly those involved in leukocyte migration and cytokine-cytokine receptor interactions. These findings indicate that needle-free vaccination was associated with distinct PCV2-specific antibody responses and local immune microenvironment changes, supporting its potential as an alternative vaccine delivery strategy for swine PCV2 vaccination.

## Simple Summary

Vaccination against porcine circovirus type 2 (PCV2) is essential for protecting pigs against PCV2-associated diseases. Needle injection, however, may cause pain, tissue damage, needle breakage, and cross-contamination between animals. In this study, we evaluated a needle-free jet injection system as an alternative vaccination method in pigs. Compared with conventional needle-based injection, high-pressure needle-free jet injection through the skin elicited higher PCV2-specific antibody levels at 0.75 mL and 0.50 mL vaccine volumes, preserved dermal collagen fiber architecture at the injection site, and was associated with distinct local immune-related responses. These findings suggest that needle-free jet injection may improve PCV2-specific immune responses and provide a potential strategy for optimizing vaccine delivery and antigen utilization.

## 1. Introduction

The skin is the largest immune organ in mammals and represents an important interface between the host and the external environment, providing both physical protection and immune surveillance [1]. The number of resident immune cells in the dermis is approximately double that of the circulatory system [2,3,4]. Epidermis-specific Langerhans cells and dermal resident dendritic cells capture foreign antigens and drain them via the extensive vascular and lymphatic networks in the dermis to the relevant lymph nodes, thereby inducing adaptive immunity [5,6]. Thus, the skin possesses the capability to activate both innate and adaptive immunity, making it a promising site for vaccine administration.

Vaccination plays a central role in the prevention and control of infectious pathogens in livestock [7]. Conventional needle-based vaccination, including intramuscular, intradermal, and subcutaneous injection, may cause injection-site tissue injury and increase the risk of cross-contamination between animals [8,9,10]. In food-producing animals, injection-site lesions may also compromise carcass quality and result in economic losses. Furthermore, although intradermal vaccination effectively leverages the skin’s unique immunological characteristics, its broad field adoption in livestock production remains constrained by technical precision requirements and the imperative for cost-effective administration [11]. Therefore, alternative vaccine delivery approaches that improve injection safety while optimizing efficient antigen delivery to the skin have attracted increasing interest [12,13].

Jet injection, a type of liquid needle-free injection delivery system, uses mechanical or high-pressure gas as the power source [14,15]. Through energy conversion, it ejects liquid medication through a nozzle, creating a jet stream that penetrates the skin and delivers the formulation into the target tissue [16,17]. Needle-free vaccine delivery may reduce injection-associated risks and has been reported to induce distinct local immune responses compared with conventional needle-based administration [9,18]. This technology has been preliminarily applied in both human and veterinary medicine.

Pigs are important livestock species that contribute substantially to global meat production and are also widely used in agricultural and biomedical research. Their anatomical, physiological, immunological, and genetic similarities to humans make them valuable large-animal models for studying infectious pathogens, vaccine responses, and other biomedical conditions [19,20,21]. Because infectious pathogens in pigs can negatively affect animal health, productivity, and farm profitability, effective vaccination is essential for preventing infectious diseases and supporting sustainable swine production [22]. However, conventional needle-based vaccination may cause injection-site pain and tissue injury, raising additional animal-welfare concerns. Porcine circovirus type 2 (PCV2) is a significant pathogen affecting swine health and causing considerable economic losses in the pig industry. The needle injection of inactivated PCV2 vaccine has also been recognized for its potential application value [23,24]. Nevertheless, the effects of different vaccine volumes delivered by needle-free injection on PCV2-specific antibody responses and local skin reactions in pigs remain insufficiently understood. In addition, the transcriptional characteristics of skin responses induced by needle-free and needle injection have not been fully characterized.

In this study, all pigs received the same inactivated PCV2 vaccine formulation from the same batch. The vaccine was administered either by needle-free injection or by needle injection. The needle-free groups received different vaccine volumes of 1.0, 0.75, 0.50, or 0.25 mL, whereas the needle injection group received 1.0 mL of the same vaccine. This design enabled comparison of the effects of vaccine volume and delivery method on PCV2-specific antibody responses and local tissue reactions.

The primary systemic outcome evaluated in this study was the PCV2-specific antibody response, which was measured by an enzyme-linked immunosorbent assay (ELISA) in serum samples collected on days 7, 14, and 28 post-immunization. Immunohistochemistry was performed to characterize changes in dendritic cell-associated markers and other stromal cell-associated signals within the skin. Additionally, samples from the injection sites were collected on day 1 after needle injection and on days 1 and 7 after needle-free injection for transcriptome sequencing. Transcriptomic analysis was further performed to characterize the molecular responses of the skin following different delivery approaches and sampling times. This study aimed to characterize the effects of different vaccine volumes and delivery methods on PCV2-specific antibody responses, local skin reactions, and skin-associated transcriptional profiles in pigs.

## 2. Materials and Methods

### 2.1. Animals

A total of 30 PCV2 antibody-negative, 22–23-day-old Duroc × (Landrace × Large White) (DLY) three-way crossbred commercial pigs were enrolled in this study. The pigs had an initial body weight of 7.14 ± 0.63 kg. The experiment was conducted in two cohorts. The two cohorts received the same vaccine formulation and injection procedures.

Eighteen pigs were randomly allocated into six groups (*n* = 3 per group) to compare immune responses following different vaccine administration volumes. The groups included a non-vaccinated control group (N), a needle injection group receiving 1.0 mL vaccine volume, and four needle-free injection groups receiving 1.0 mL, 0.75 mL, 0.50 mL or 0.25 mL vaccine volumes. The inactivated PCV2 vaccine was administered into the lateral cervical skin region using either needle injection or the POK-MBX JNFI jet needle-free injection system. For needle injection, the vaccine was manually delivered using a disposable sterile 5 mL syringe equipped with a 23 G needle (0.6 mm × 25 mm; Anhui Kangtai Medical Treatment Instrument Co., Ltd., Hefei, China) at an approximately 45° insertion angle. For needle-free injection, the POK-MBX JNFI system was operated at a working pressure of 0.60 MPa according to the manufacturer’s recommended procedures, with the entire vaccine volume administered in a single shot. All injections were performed according to the assigned experimental groups. Blood samples were collected on days 7, 14, and 28 after vaccination for analysis of PCV2-specific antibody responses.

**Figure 1:**
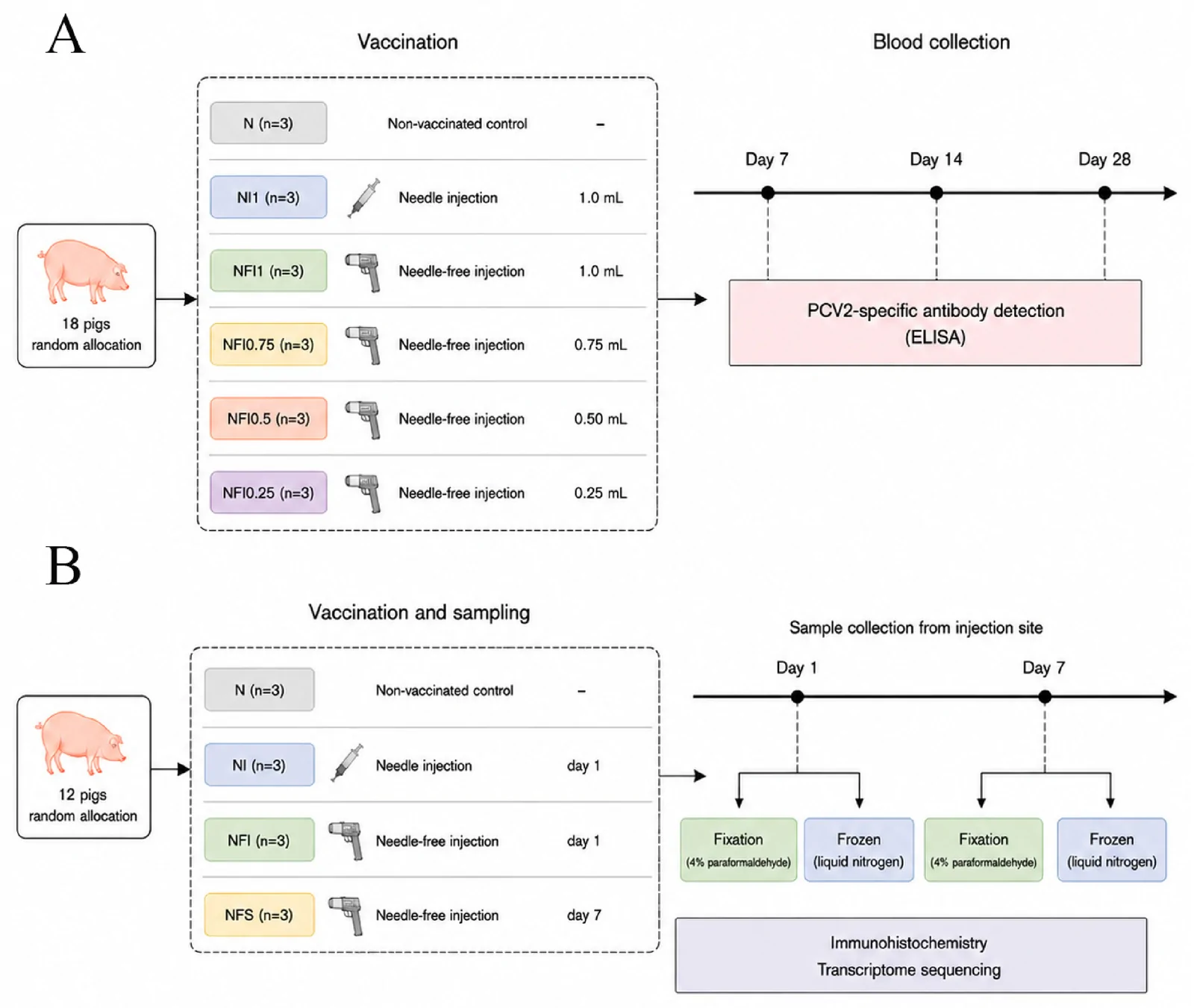
Experimental design and sampling schedule. (**A**) Eighteen pigs were used to evaluate PCV2-specific antibody responses after vaccination with different injection methods and vaccine volumes. (**B**) Twelve additional pigs were used to assess injection-site responses by immunohistochemistry and transcriptome sequencing. Tissue samples were either fixed in 4% paraformaldehyde or frozen in liquid nitrogen. N: blank control group; NI1: 1.0 mL needle injection group; NF1: 1.0 mL needle-free injection group; NF0.75: 0.75 mL needle-free injection group; NF0.5: 0.50 mL needle-free injection group; NF0.25: 0.25 mL needle-free injection group; NI: needle injection; NFI: needle-free injection; NFS: needle-free injection sampled on day 7.

Twelve pigs were randomly allocated into four groups (*n* = 3 per group): a control group (N), a group sampled 1 day after needle injection (NI), and two groups sampled 1 day (NFI) and 7 days (NFS) after 1.0 mL needle-free injection. Samples obtained were partially fixed in 4% paraformaldehyde solution and the remaining samples were immediately frozen in liquid nitrogen (Figure 1).

### 2.2. Major Reagents and Instruments

A commercial baculovirus-expressed inactivated porcine circovirus type 2b (PCV2b) subunit vaccine (strain CP08, Cap protein; Yuanlikang, Sinopharm Animal Health Co., Ltd., Wuhan, China); PCV2 antibody ELISA detection kit (BioChek, Cat. No. SK105, Reeuwijk, The Netherlands); total RNA extraction and reverse-transcription reagents (Vazyme Biotech Co., Ltd., Nanjing, China, Cat. No. R323); real-time qPCR kit (Vazyme Biotech Co., Ltd., Nanjing, China, Cat. No. Q511); NEBNext Ultra II RNA Library Prep Kit for Illumina (New England Biolabs, Ipswich, MA, USA); DEPC-treated water (Saiweier Biotechnology Co., Ltd., Wuhan, China); chloroform; and isopropanol (Sinopharm Chemical Reagent Co., Ltd., Shanghai, China) were used in this study. A high-speed tissue grinder (Servicebio, Wuhan, China), a high-speed centrifuge (Eppendorf, Hamburg, Germany), a micro-volume spectrophotometer (NanoDrop, Thermo Scientific, Wilmington, DE, USA), a real-time PCR system (QuantStudio 7, Thermo Scientific, Waltham, MA, USA), an ultrapure water system (Milli-Q, Merck, Darmstadt, Germany), a microplate reader (Tecan, Männedorf, Switzerland), and a conventional thermal cycler (Biometra, Göttingen, Germany) were used in this study.

### 2.3. ELISA

The serum samples were analyzed for IgG antibodies against PCV2 using an indirect ELISA with the PCV2 Antibody Test Kit (SK105; BioChek, Reeuwijk, The Netherlands), following the manufacturer’s protocol. In brief, the serum was diluted with the provided diluent and added to PCV2 antigen-coated microtiter plates. After incubation at room temperature and washing, an anti-swine conjugate (alkaline phosphatase in Tris buffer) was added and incubated again. Following a second wash, p-Nitrophenyl Phosphate (pNPP) substrate was added. The reaction was stopped after a 15 min incubation at room temperature by adding stop solution. Optical density (OD) was measured at 405 nm using a monochromatic ELISA reader (Tecan, Männedorf, Switzerland). Results were calculated as S/P ratios, with a cut-off for negative samples at an S/P ratio of 0.49 or less.

### 2.4. Immunohistochemistry

Immunohistochemical analysis was performed to characterize local tissue architecture and the distribution of antigen-presenting cell-associated markers at the injection sites following needle-free and needle injection.

Paraffin sections (7 μm) were deparaffinized in xylene and rehydrated through a graded ethanol series. Endogenous peroxidase activity was blocked with 3% hydrogen peroxide at 37 °C for 10 min. Antigen retrieval was performed using proteinase K at 37 °C for 10 min. After blocking with 5% bovine serum albumin (BSA, Bioss, Beijing, China) for 40 min, sections were incubated overnight at 4 °C with rabbit anti-SIRP-alpha (Proteintech, Wuhan, China, 1:100), rabbit anti-CD11c (Proteintech, Wuhan, China, 1:100), rabbit anti-Langerin (Proteintech, Wuhan, China, 1:100), or mouse anti-Vimentin antibodies (Boster, Wuhan, China, 1:100). For negative controls, PBS was used in place of the primary antibodies. The following day, sections were incubated with HRP-conjugated goat anti-rabbit IgG or HRP-conjugated goat anti-mouse IgG, as appropriate, at 37 °C for 1 h. Immunoreactivity was visualized using DAB, and nuclei were counterstained with hematoxylin. Finally, sections were dehydrated, cleared, mounted with neutral balsam, and scanned using a slide scanner (Grundium Oy, Tampere, Finland).

### 2.5. RNA-Seq

RNA sequencing was performed to characterize the local transcriptional responses in skin tissue following needle-free and needle-based injection and to identify biological pathways associated with the different local tissue responses.

#### 2.5.1. Extraction of Skin Tissue RNA

Total RNA was extracted from skin samples of each group according to the reagent manufacturer’s instructions. The quality of the extracted RNA was assessed using a micro-spectrophotometer (NanoDrop, Thermo Scientific, Wilmington, DE, USA) and agarose gel electrophoresis.

#### 2.5.2. cDNA Library Construction

The extracted RNA was used to construct cDNA libraries following the reagent manufacturer’s instructions. Oligo(dT) magnetic beads were used to enrich mRNA with poly(A) tails from the total RNA. The enriched mRNA was randomly fragmented using Fragmentation Buffer. Random oligonucleotide primers were then used to synthesize the first strand cDNA using the fragmented mRNA as a template. The second strand cDNA was synthesized using DNA polymerase I and RNase H. After purification, the cDNA underwent end repair, addition of an A-tail at the 3' end, and ligation with sequencing adapters. AMPure XP beads (Beckman Coulter Life Sciences, Indianapolis, IN, USA) were used to select 400–500 bp cDNA fragments, which were then subjected to PCR amplification and further purified using AMPure XP beads to obtain the final cDNA library.

#### 2.5.3. Transcriptome Sequencing Analysis and Quality Control

The quality of the constructed cDNA libraries was assessed using an Agilent 2100 Bioanalyzer (Agilent Technologies, Waldbronn, Germany). Libraries that passed quality control were sequenced on an Illumina HiSeq X platform (Illumina, San Diego, CA, USA) to generate raw reads. FastQC v0.11.8 was used to filter out low-quality reads and reads containing adapters. HISAT2 v2.0.5 was used to align the reads to the pig (*Sus scrofa*) reference genome (http://asia.ensembl.org/Sus_scrofa/Info/Annotation(accessed on 1 July 2022)). Post-alignment, HTSeq v0.9.1 was used for assembly and quantification of the aligned reads.

#### 2.5.4. Normalization of Sequencing Data

Normalization of the read counts corresponding to genes was performed using the Fragments Per Kilobase of transcript per Million mapped reads (FPKM) method. For a given gene, the FPKM value was calculated by dividing the number of reads mapped to the gene by the total length of the exons (in kilobases) and then dividing by the total number of mapped reads (in millions). The formula is as follows:

$$FPKM=\frac{Total\;Exon\;Fragments}{Mapped\;Reads\left(Millions\right)\;\times \;Exon\;Length\;(Kb)}$$

### 2.6. Bioinformatics Analysis

#### 2.6.1. Differential Gene Expression Screening

Differentially expressed genes (DEGs) were identified using the DESeq2 package. Genes with |log_2_FoldChange| > 2 and *p* < 0.05 were defined as DEGs.

#### 2.6.2. Functional Annotation of Differentially Expressed Genes

Functional annotation of DEGs was performed using the Gene Ontology (GO) and Kyoto Encyclopedia of Genes and Genomes (KEGG) databases. GO annotations include molecular function (MF), cellular component (CC), and biological process (BP). KEGG database provides a systematic analysis of gene metabolic and signaling pathways.

#### 2.6.3. Gene Set Enrichment Analysis (GSEA)

GSEA ranks all detected genes based on their correlation with phenotypes, enabling enrichment analysis without predefining differentially expressed genes (DEGs), thus reducing false negatives due to inconsistent DEG thresholds. GSEA quantifies enrichment scores to determine the contribution of gene sets to specific phenotypes. In this study, genes between groups were ranked by log_2_FoldChange, using the KEGG database as the preset gene set. Enrichment scores were calculated with the GSEA approach, providing a normalized enrichment score (NES). Pathways with |NES| > 1, *p* < 0.05, and FDR < 0.25 were considered significantly enriched. The FDR threshold applied in GSEA differs from that used for DEG-based enrichment analysis because GSEA evaluates pathway-level coordinated gene expression patterns rather than individual gene-level differential expression.

#### 2.6.4. Enrichment Analysis of Differentially Expressed Genes

GO enrichment was performed using the clusterProfiler package (v4.4.4), with enrichment analysis algorithms applying hypergeometric tests and Benjamini–Hochberg correction to determine the potential biological functions of DEGs between groups. GO terms with *p* < 0.05 and FDR < 0.05 were considered significantly enriched among DEGs. A more stringent FDR threshold was applied for DEG-based enrichment analysis because this analysis was based on individual differentially expressed genes.

### 2.7. qRT-PCR

Total RNA was extracted from 100 mg skin samples using TRIzol, homogenized with zirconium beads, incubated with chloroform, and centrifuged. The aqueous phase was precipitated with isopropanol, washed with ethanol, and dissolved in DEPC water (Servicebio, Wuhan, China). Then, 1 μg RNA was treated with gDNA wiper Mix (Vazyme, Nanjing, China), and reverse-transcribed using HiScript II qRT SuperMix II (Vazyme, Nanjing, China). Primers were designed using NCBI Primer Blast and synthesized by Genscript, Nanjing, China. The PCR reaction mixture (20 μL) contained 10 μL 2× AceQ qPCR SYBR Green Master Mix (Vazyme, Nanjing, China), 0.4 μL each of forward and reverse primers, 1 μL cDNA, 0.4 μL 50× ROX Dye, and Milli-Q water. PCR was performed with the following program: initial denaturation at 95 °C for 5 min, followed by 40 cycles of denaturation at 95 °C for 10 s, and annealing/extension at 60 °C for 30 s. Relative quantification was performed using the 2^−ΔΔCt^ method, with *GAPDH* as the reference gene (Table S1).

### 2.8. Statistical Analysis

All data were analyzed using R software (v4.2.1) for one-way ANOVA followed by LSD multiple comparison tests with Bonferroni correction. Data visualization was performed with GraphPad Prism 8.0, and results were expressed as mean (SD). Differences between groups were considered significant at *p* < 0.05 and highly significant at *p* < 0.01.

## 3. Results

### 3.1. PCV2-Specific Antibody Responses Following Different Vaccine Volumes and Delivery Methods

During the observation period, all pigs survived, and no abnormalities in activity or appetite were observed after vaccination. A small localized wheal was observed at the injection sites in both the needle-free injection and needle-based injection groups, which gradually subsided without intervention.

**Figure 2:**
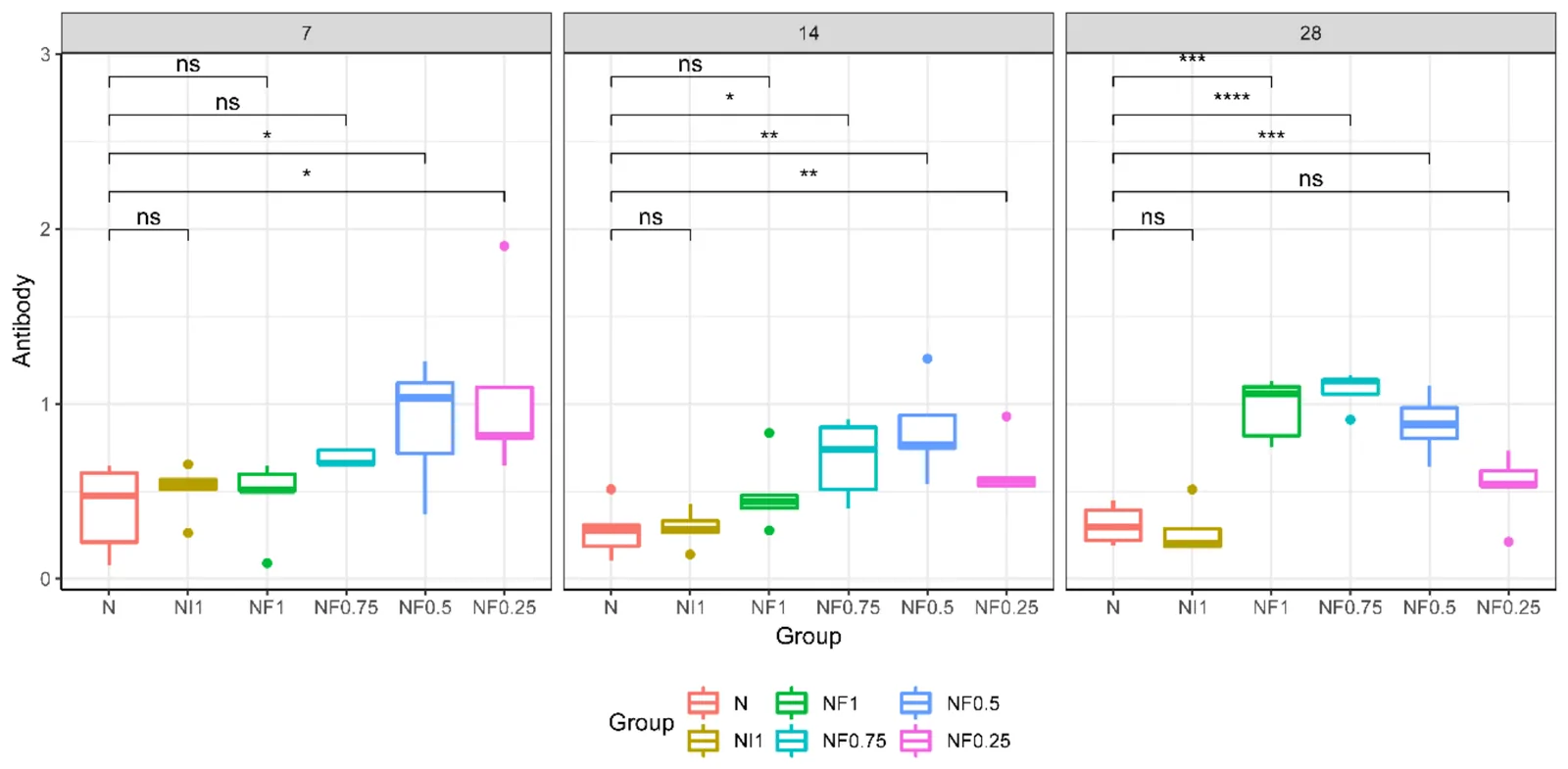
Serum PCV2-specific antibody levels in pigs at 7, 14, and 28 days post-vaccination, determined by indirect ELISA. N: blank control group; NI1: 1.0 mL needle injection group; NF1: 1.0 mL needle-free injection group; NF0.75: 0.75 mL needle-free injection group; NF0.5: 0.50 mL needle-free injection group; NF0.25: 0.25 mL needle-free injection group. *n* = 3 pigs per group. ns: no significant difference; * *p* < 0.05; ** *p* < 0.01; *** *p* < 0.001; **** *p* < 0.0001.

Serum PCV2-specific antibody levels were measured on days 7, 14, and 28 after vaccination. Under the conditions evaluated in this study, the 1.0 mL needle-injection group did not differ significantly from the non-vaccinated control group in PCV2-specific antibody levels at days 7, 14, or 28 (Figure 2). This result should be interpreted cautiously, as the relatively large injection volume and differences in delivery technique may have influenced the observed antibody response.

Among the needle-free groups, the 0.50 and 0.25 mL groups showed significantly higher antibody levels than the non-vaccinated group on days 7 and 14 (*p* < 0.05), while the 0.75 mL group showed a significant increase by day 14 (*p* < 0.05). By day 28, the 1.0, 0.75, and 0.50 mL needle-free groups showed significantly higher PCV2-specific antibody levels than both the 1.0 mL needle-injection and non-vaccinated groups (*p* < 0.01), whereas the 0.25 mL group did not differ significantly from either group.

Thus, under the experimental conditions of this study, the 0.50–1.0 mL needle-free groups showed significantly higher PCV2-specific antibody levels than the 1.0 mL needle-injection group by day 28. These findings should be interpreted in the context of the different delivery methods and vaccine volumes evaluated.

### 3.2. Needle-Free Injection Is Associated with Distinct Distributions of Antigen-Presenting Cell-Associated Signals in the Skin

**Figure 3:**
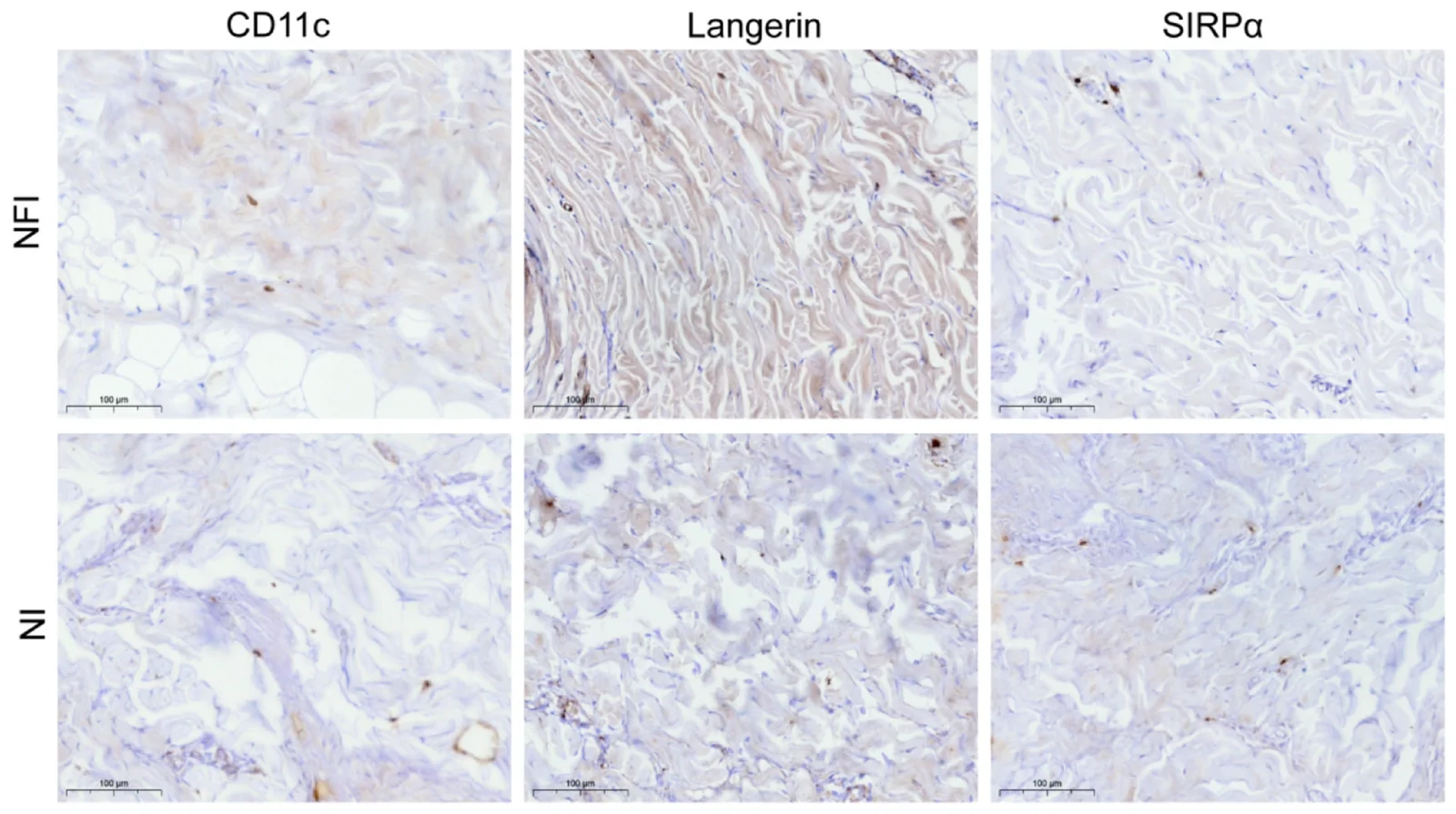
Distinct local distributions of antigen-presenting cell-associated markers following needle-free and needle injection. Scale bar = 100 µm.

Immunohistochemical staining was performed to characterize local tissue changes and the distribution of antigen-presenting cell-associated markers at the injection sites in the NFI and NI groups. In the NI group, antigen-presenting cell-associated signals were mainly observed around blood and lymphatic vessels, accompanied by disrupted dermal collagen organization. In contrast, the dermal collagen fiber architecture was better preserved in the NFI group. Dendritic cell-associated signals exhibited a more dispersed distribution within the dermal collagen matrix, rather than the perivascular accumulation observed in the NI group. Langerin-positive cells were less frequently detected in the dermis of the NFI group (Figure 3).

**Figure 4:**
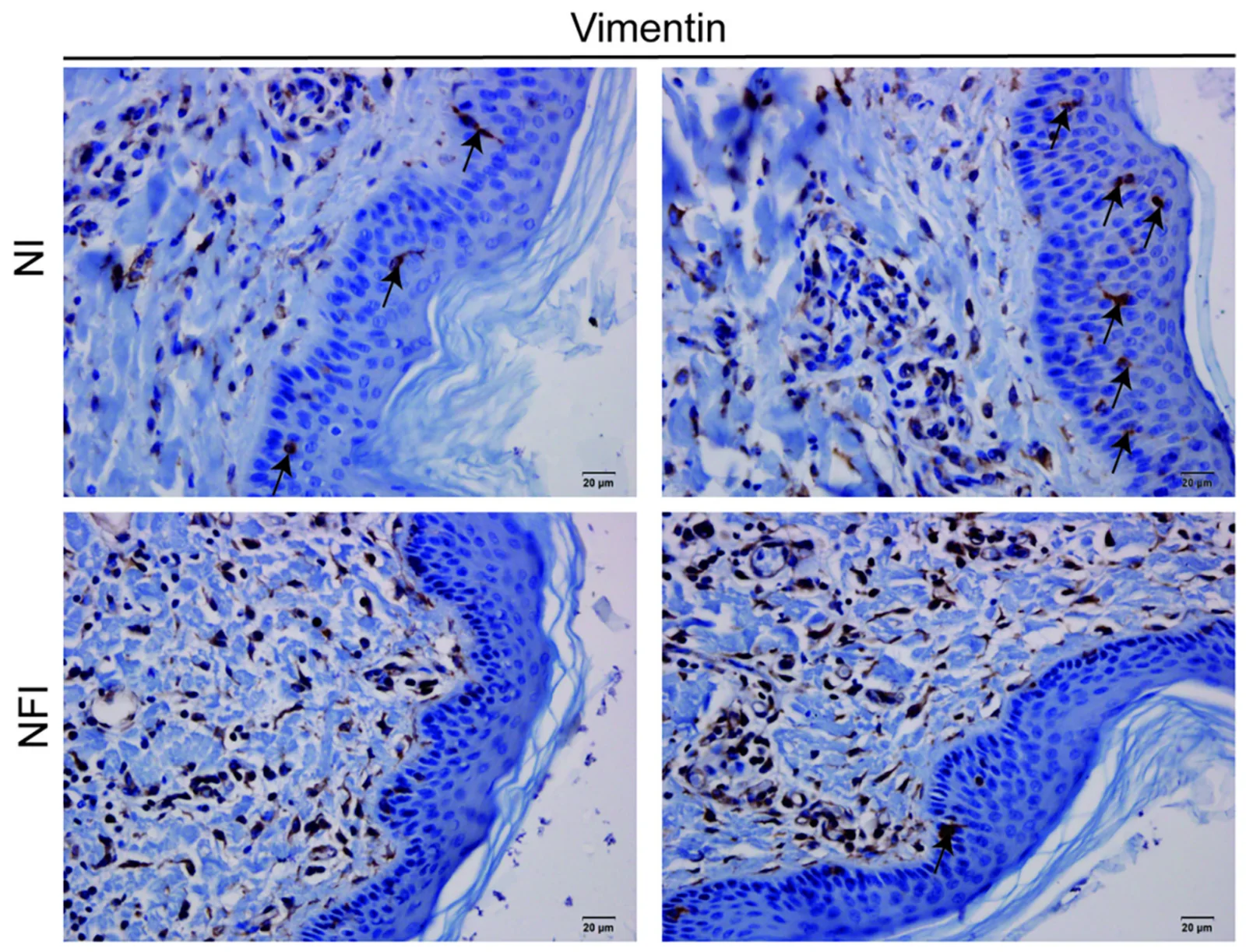
Representative immunohistochemical images of Vimentin expression in the skin at the injection sites in the NI and NFI groups. Brown indicates positive Vimentin immunoreactivity, while blue staining represents cell nuclei counterstained with hematoxylin. Black arrows point to Vimentin-positive cells. Scale bar = 20 µm.

Vimentin staining was further performed to characterize the distribution of Vimentin-positive cells in the epidermal and dermal compartments. In the NI group, numerous Vimentin-positive cells with dendritic-like morphology were observed mainly in the stratum granulosum and stratum spinosum. In comparison, fewer Vimentin-positive cells with dendritic-like morphology were observed in the epidermis of the NFI group, with Vimentin-positive cells mainly distributed in the stratum basale and underlying dermis (Figure 4).

### 3.3. Quality Control of Sequencing Data and Gene Expression Distribution

The 12 skin tissue samples collected from the injection sites generated high-quality sequencing data, with Q30 values above 91.83% and mapping rates above 95.14% (Table S2), supporting the reliability of the transcriptomic analyses.

**Figure 5:**
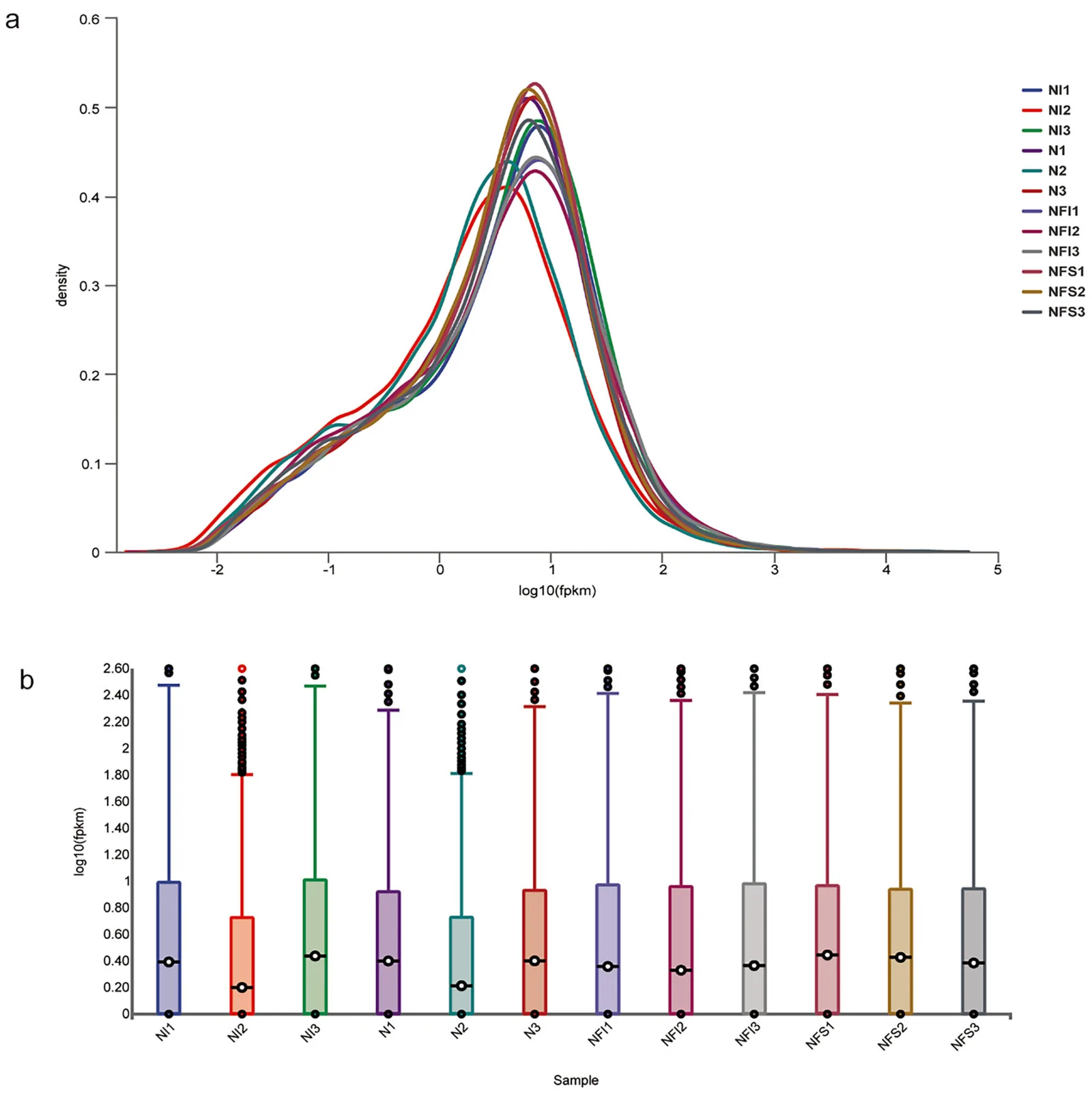
Distribution of gene expression levels across samples. (**a**) Density distribution of log_10_ (FPKM) values for individual samples (colored lines represent different biological replicates); (**b**) Box plots of log_10_ (FPKM) values across all samples. The *X-axis* denotes sample groups, and the *Y-axis* represents log_10_ (FPKM).

According to the FPKM density distribution plot of the sequencing data (Figure 5a), the FPKM density distribution trends of the 12 samples were roughly similar. Most genes exhibited medium expression levels, while a small portion showed low or high expression levels, spanning the range of 10^2^ to 10^4^. Additionally, the median FPKM values of gene expression levels across all samples were roughly equal, with no significant outliers or anomalies (Figure 5b).

### 3.4. Cluster Analysis, Identification and Analysis of Differentially Expressed Genes

**Figure 6:**
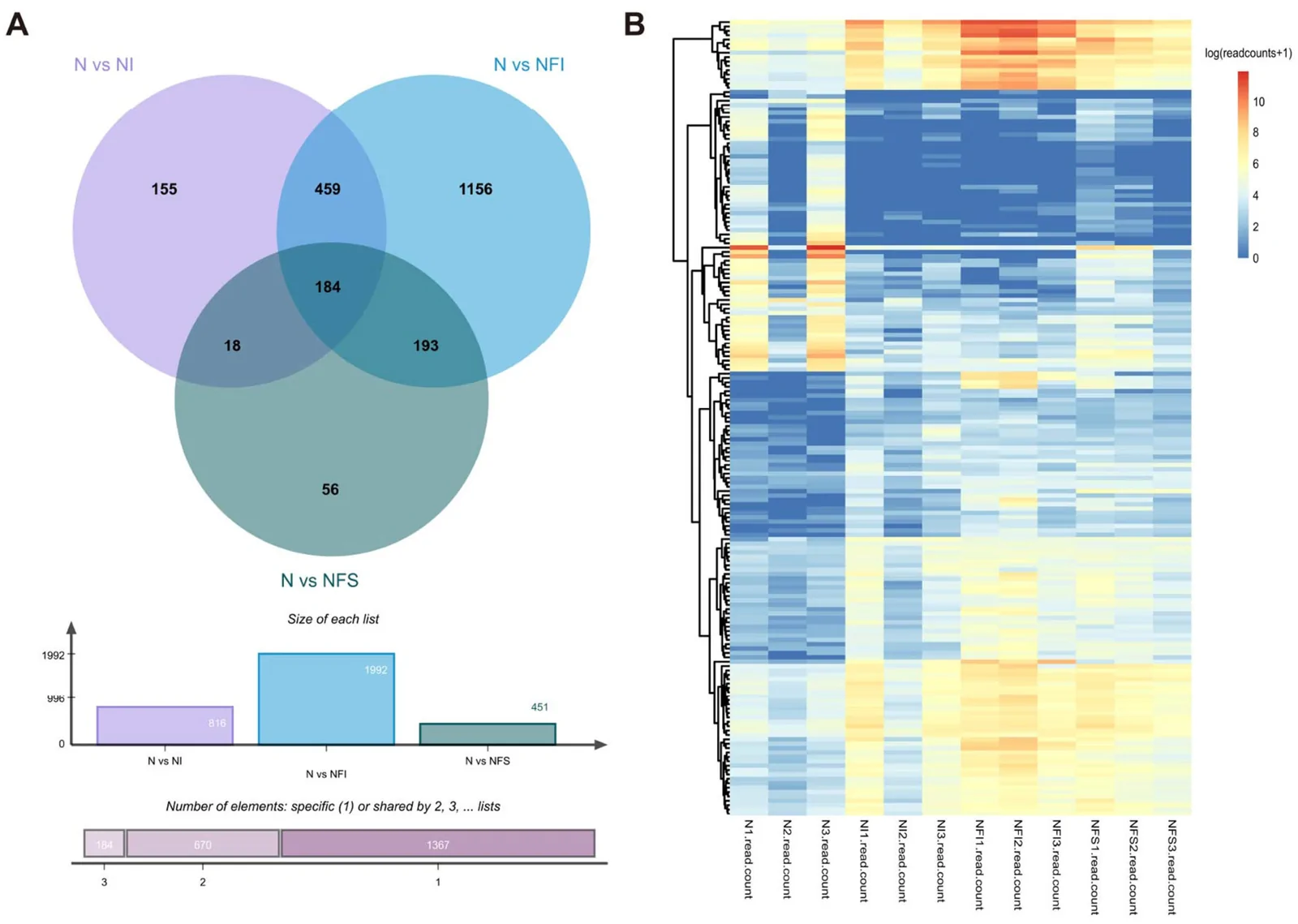
Venn diagram and clustering heat map of differentially expressed gene distribution. (**A**) Venn plot of differentially expressed gene distribution. (**B**) Heat map of clustering of whole gene expression patterns for each sample.

Differentially expressed genes (DEGs) were screened between groups using the criteria of |log_2_FoldChange| > 2 and *p* < 0.05. Comparisons of the N group with the NI, NFI, and NFS groups identified 816, 1992, and 451 DEGs, respectively. Among these, 532, 1100, and 301 genes were upregulated, whereas 284, 893, and 150 genes were downregulated, respectively. The Venn diagram showed that 184 DEGs were shared among the three comparisons (Figure 6a).

Heatmap cluster analysis of gene expression (Figure 6b) suggested that samples within the same group exhibited similar expression patterns, confirming the good reproducibility of the sequencing experiment and the reliability of the differentially expressed genes (DEGs).

### 3.5. GSEA

**Figure 7:**
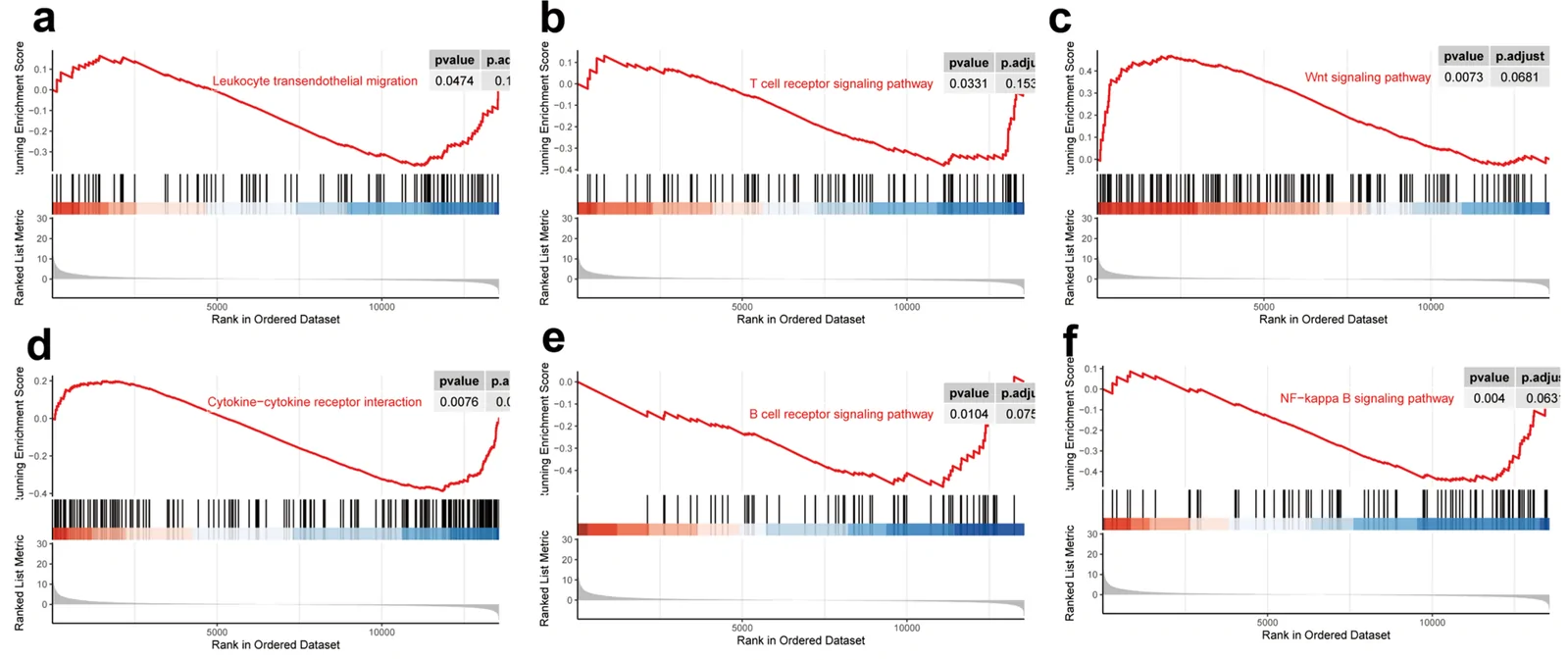
GSEA between NI group and N group, including (**a**) leukocyte transendothelial migration pathway, (**b**) T-cell receptor signaling pathway, (**c**) Wnt signaling pathway, (**d**) cytokine–cytokine receptor interaction, (**e**) B-cell receptor signaling pathway, and (**f**) NF-κB signaling pathway. The x-axis represents the ranked gene list according to differential expression statistics, and the y-axis represents the running enrichment score (ES). The red curve indicates the running ES across the ranked gene list. The vertical black bars represent the positions of genes belonging to the corresponding pathway within the ranked gene list. NES, normalized enrichment score. *p* value and *p*.adjust represent the statistical significance of pathway enrichment.

Using the log_2_FoldChange values from differential expression analysis as the differential metric ranking file, GSEA was performed on the KEGG pathways. The results indicated that, compared to the N group (Figure 7), the NI group showed significant enrichment in pathways such as leukocyte transendothelial migration (NES = −1.38, *p* < 0.05, FDR < 0.25), T-cell receptor signaling pathway (NES = −1.42, *p* < 0.05, FDR < 0.25), cytokine–cytokine receptor interaction (NES = −1.60, *p* < 0.01, FDR < 0.25), B-cell receptor signaling pathway (NES = −1.61, *p* < 0.05, FDR < 0.25), and NF-κB signaling pathway (NES = −1.67, *p* < 0.01, FDR < 0.25). The NI treatment suppressed the expression of most related genes in these pathways. In contrast, the Wnt signaling pathway (NES = 1.53, *p* < 0.01, FDR < 0.25) was significantly activated after NI treatment.

**Figure 8:**
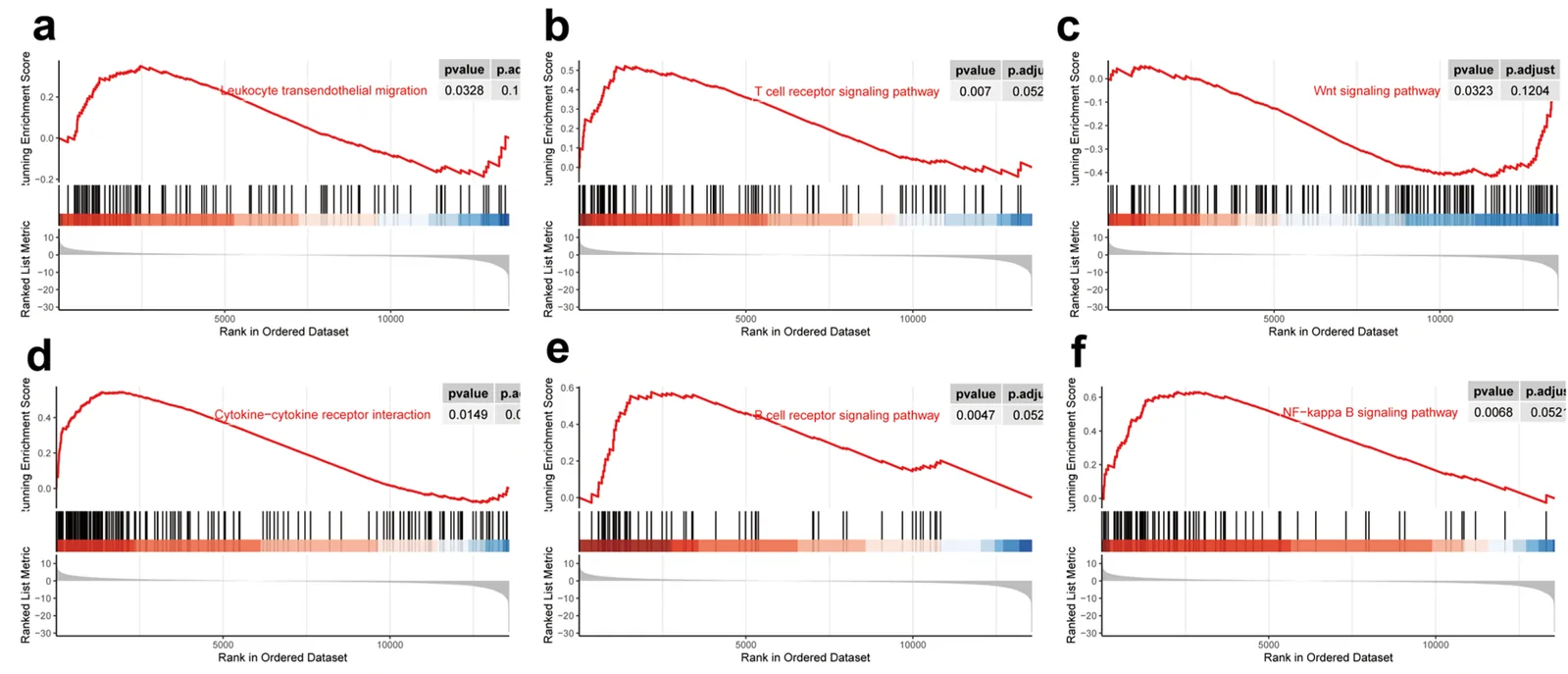
Gene set enrichment analysis between NFI group and N group, including (**a**) leukocyte transendothelial migration pathway, (**b**) T-cell receptor signaling pathway, (**c**) Wnt signaling pathway, (**d**) cytokine–cytokine receptor interaction, (**e**) B-cell receptor signaling pathway, and (**f**) NF-κB signaling pathway. The x-axis represents the ranked gene list according to differential expression statistics, and the y-axis represents the running enrichment score (ES). The red curve indicates the running ES across the ranked gene list. The vertical black bars represent the positions of genes belonging to the corresponding pathway within the ranked gene list. NES, normalized enrichment score. *p* value and *p*.adjust represent the statistical significance of pathway enrichment.

In the comparison between the NFI and N groups (Figure 8), pathways such as leukocyte transendothelial migration (NES = 1.37, *p* < 0.05, FDR < 0.25), T-cell receptor signaling pathway (NES = 2.04, *p* < 0.01, FDR < 0.25), cytokine–cytokine receptor interaction (NES = 2.37, *p* < 0.05, FDR < 0.25), B-cell receptor signaling pathway (NES = 2.05, *p* < 0.01, FDR < 0.25), and NF-κB signaling pathway (NES = 2.44, *p* < 0.01, FDR < 0.25) were significantly enriched and tended to be activated in the NFI group. Conversely, the Wnt signaling pathway (NES = −1.34, *p* < 0.05, FDR < 0.25) exhibited a suppressed profile after NFI treatment.

### 3.6. GO and KEGG Enrichment for DEGs

Enrichment analysis of DEGs revealed the top 10 GO terms with the smallest FDR values for each group comparison (or all significant GO terms if fewer than 10 were significant). The FDR represents the adjusted *p*-value after multiple hypothesis testing; the smaller the FDR, the higher the confidence in the enrichment of DEGs in the relevant GO terms.

**Figure 9:**
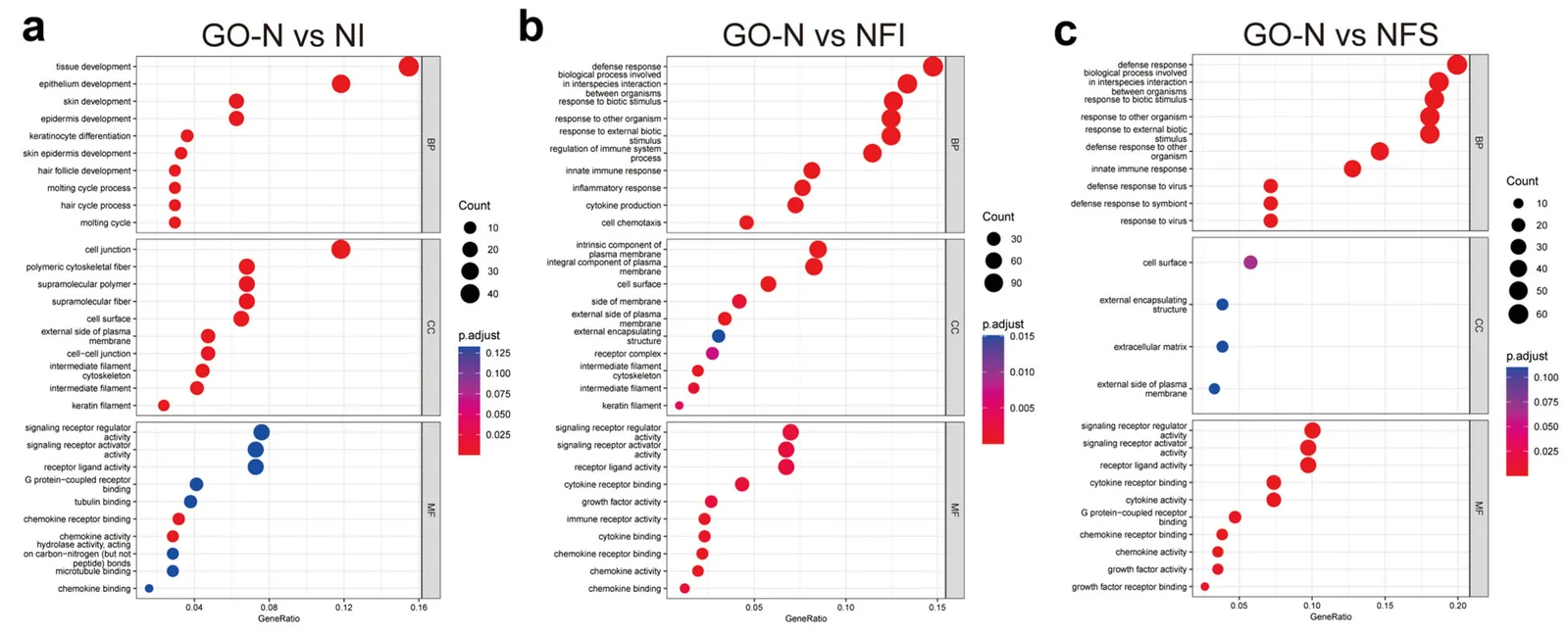
Results of GO enrichment analysis of differential genes between NI, NFI, NFS groups and N groups. (**a**) Results of GO enrichment analysis of differentially expressed genes in NI group and N group. (**b**) Results of GO enrichment analysis of differentially expressed genes in NFI group and N group. (**c**) Results of GO enrichment analysis of differentially expressed genes in NFS group and N group; the horizontal coordinate indicates the enrichment factor, which is the ratio of the proportion of differential genes annotated to a GO term pathway to the genes annotated to that GO term in all genes, and the vertical coordinate indicates the GO term name. BP: biological process, CC: cellular component, MF: molecular function.

GO enrichment analysis (Figure 9) indicated that, between the N and NI groups, GO terms related to skin regeneration such as tissue development, epithelium development, skin development, epidermis development, and cell junction were significantly enriched. Conversely, between the N and NFI groups and the N and NFS groups, GO terms related to immune activities, including defense response biological process, innate immune response, cytokine production, and chemokine activity, were significantly enriched.

### 3.7. Real-Time Quantitative PCR Validation of Differentially Expressed Genes

To further validate the RNA-seq findings, DEGs involved in immune-related pathways identified by GO/KEGG enrichment analysis were selected for qRT-PCR validation. Candidate genes were selected based on their association with cytokine signaling, chemokine activity, leukocyte migration, and immune cell regulation. The selected genes included *IL6*, *IL7*, *IL8*, *IL10*, *CXCL9*, *CXCL10*, *CXCL11*, *CCL2*, *CCL4*, *CCL5*, *CCL8*, *CCL19*, *XCL1*, *CXCL2*, *CXCL6*, *CXCL14*, *CCRL2*, *CCR1*, *CCR5*, *CCR7*, *CCR9*, *ACKR3*, *ACKR4*, *ICOS*, *INOS*, *ICAM1*, and *PPBP.* The qRT-PCR expression patterns were generally consistent with the RNA-seq results, confirming the reliability of the transcriptomic analysis. The qRT-PCR results also indicated that in the NI group, except for *CXCL6* (*p* < 0.05) and *CCR7* (*p* < 0.01), the expression levels of the other genes did not show significant differences compared to the N group (Figures S1 and S2). In contrast, NFI treatment induced significant upregulation of immune-related genes compared with the N group, including genes involved in chemokine signaling and cytokine regulation. The qRT-PCR results also indicated that, in the NFS group, except for *CXCL14* (*p* > 0.05), *CCR1* (*p* > 0.05), and *IL10* (*p* > 0.05), the expression levels of the other genes were significantly lower than those in the NFI group. Furthermore, except for *XCL1* (*p* < 0.01), *IL10* (*p* < 0.05), *IL6* (*p* < 0.01), *CCRL2* (*p* < 0.01), *CCR9* (*p* < 0.01), *CCL4* (*p* < 0.01), and *CCR1* (*p* < 0.01), the expression levels of the remaining genes did not show statistical differences compared to the N group (Figures S3 and S4).

## 4. Discussion

### 4.1. Enhanced Immunogenicity and Reduced Vaccine Volume with Needle-Free PCV2 Vaccination

In this study, needle-free injection showed potential for volume reduction under the conditions tested for inactivated PCV2 vaccination in pigs. Although intradermal vaccination can effectively induce immune responses, its efficacy and animal tolerance may be affected by needle characteristics, injection conditions, vaccine properties, and needle-associated pain or tissue damage [25,26]. Needle-free injection represents an alternative transdermal delivery approach that uses high-pressure liquid jets to deposit vaccines within the skin or underlying tissues, potentially improving antigen distribution while reducing needle-associated limitations [27,28]. Under the conditions of this study, needle-free administration at vaccine volumes of 0.50 and 0.75 mL induced higher PCV2-specific antibody responses than 1.0 mL needle injection. However, this finding does not indicate an intrinsic limitation of the intradermal route. Previous studies have shown that PCV2 vaccines can induce immune responses when delivered intradermally using dedicated delivery systems with smaller volumes. Therefore, the observed difference may reflect the combined effects of delivery technique, injection volume, and local antigen distribution. These findings suggest that the higher antibody responses observed with needle-free administration may be associated with differences in antigen delivery and local tissue responses; however, the underlying mechanisms require further investigation.

### 4.2. Needle-Free Injection Preserves Dermal Architecture and Is Associated with Distinct Local Tissue Responses

The skin contains abundant antigen-presenting cells, including epidermal Langerhans cells and dermal dendritic cells, which are important for antigen recognition and immune initiation [29]. In pigs, dermal dendritic cells express markers such as CD11c, Langerin, and SIRP, while Vimentin expression has been used as an indicator for epidermal Langerhans cells, providing valuable approaches for characterizing immune cell distribution within porcine skin [29,30,31,32,33,34].

In the present study, immunohistochemical analysis revealed distinct tissue responses following needle-free and needle injection. Compared with needle injection, needle-free injection better preserved dermal collagen architecture and resulted in a more dispersed distribution of antigen-presenting cell-associated signals within the dermal matrix. These findings may be related to the distinct tissue deformation and intradermal distribution patterns produced by liquid-jet delivery.

The extracellular matrix, particularly collagen networks, provides structural support and regulates immune cell localization, migration, and tissue homeostasis [35,36,37]. Excessive tissue disruption may activate repair-associated responses and alter the allocation of cellular resources toward regeneration. Therefore, the preserved dermal structure observed after needle-free injection may provide a tissue environment that is more compatible with immune-cell localization and tissue homeostasis.

### 4.3. Transcriptomic Characterization of Differential Local Responses

The systemic antibody results and local tissue findings represent complementary levels of the vaccination response. Serum PCV2-specific antibodies were used as the primary systemic endpoint of immunogenicity, whereas histological, immunohistochemical, and transcriptomic analyses were performed to characterize local tissue and molecular responses associated with the different delivery approaches. These local analyses were not intended to directly establish the mechanism responsible for antibody production, but rather to characterize tissue and molecular changes that may help explain the distinct responses associated with the two administration methods.

Following tissue administration, the local microenvironment determines whether immune activation or tissue repair becomes the dominant biological response [38,39]. Skin injury repair involves coordinated interactions among keratinocytes, fibroblasts, stromal cells, and immune cells, with Wnt signaling playing an important role in tissue regeneration and homeostasis [40,41]. In the present study, transcriptomic analysis revealed different molecular responses following needle-based and needle-free injection, suggesting that these two delivery approaches may create distinct tissue states after administration.

Needle penetration induces localized tissue microdamage. Compared with the N group, the NI group showed significant activation of the Wnt signaling pathway at the injection site. The Wnt pathway plays essential roles in skin and hair follicle development, tissue regeneration, and homeostasis maintenance [42,43,44]. This finding may reflect the initiation of a tissue repair program triggered by mechanical injury [45]. Although tissue repair is critical for maintaining structural integrity, such responses may be associated with a local tissue state characterized by repair-related activity.

Compared with the N group, needle-free injection was associated with enrichment of immune-related pathways involved in leukocyte migration, T-cell receptor signaling, B-cell receptor signaling, cytokine-cytokine receptor interactions, and NF-κB activation. Unlike needle injection, needle-free delivery does not require direct tissue penetration by a needle, which may reduce structural disruption at the injection site. Meanwhile, the high-pressure jet provides rapid mechanical stimulation that may enhance innate immune sensing, promote chemokine production, facilitate leukocyte recruitment, and activate cytokine-mediated signaling pathways, including NF-κB-related responses [46,47]. However, the underlying cellular and molecular mechanisms require further experimental validation. These changes may establish a local microenvironment favorable for antigen uptake and presentation, thereby supporting subsequent T-cell- and B-cell-associated adaptive immune activation [48].

Consistently, GO enrichment analysis showed that the DEGs identified in the N versus NI, N versus NFI, and N versus NFS comparisons were mainly associated with immune-related biological processes, including cytokine production, chemokine activity, defense response, and innate immune response [49]. These findings suggest that the different injection treatments were associated with changes in immune-related processes in the local tissue compared with the non-vaccinated control at the evaluated time points.

Chemokine signaling may facilitate local immune-cell recruitment and provide a foundation for subsequent systemic immune responses. Previous studies have shown that the *CCR7*-*CCL19*/*CCL21* axis regulates the migration of antigen-presenting cells from peripheral tissues to draining lymph nodes in other immunological contexts [31,50]. However, whether this chemokine axis directly mediates antigen-presenting cell migration following needle-free vaccination remains to be experimentally validated. In the present study, cytokine- and chemokine-related genes were transiently upregulated after needle-free injection and returned to lower expression levels by day 7, suggesting that needle-free injection may induce a time-dependent and regulated local immune activation process rather than prolonged inflammatory stimulation. These transcriptomic findings characterize local molecular responses and should not be interpreted as direct evidence that these pathways caused the enhanced systemic antibody response.

Several limitations should be acknowledged. First, the small sample size (*n* = 3 per group) limits the generalizability of the findings. Second, the 1.0 mL needle-injection volume was relatively large for intradermal administration, and vaccine deposition was not directly verified; therefore, the lack of a significant antibody response should not be interpreted as evidence of vaccination failure, but rather as a finding under the specific experimental conditions. Third, skin thickness was not measured and may have affected needle-free injection performance and tissue distribution. In addition, cellular immunity and protective efficacy were not directly evaluated, and the proposed mechanisms require further validation. Finally, the absence of a time-matched needle injection group at day 7 limited the direct comparison of immune-response dynamics between the two delivery methods. Future studies incorporating larger cohorts, longitudinal sampling, skin-thickness measurements, comprehensive immune analyses, and challenge models will be required to further clarify the mechanisms and protective potential of needle-free vaccination.

### 4.4. Potential Application and Future Development

The present findings support the potential application of needle-free PCV2 vaccination in swine production. Further studies are needed to optimize delivery parameters, evaluate protective efficacy and animal welfare, and validate its performance under commercial conditions. Larger field studies and appropriate regulatory evaluation will also be required before broader national and international application.

## 5. Conclusions

In conclusion, under the conditions tested, needle-free injection elicited higher PCV2-specific antibody responses and showed dose-sparing potential in pigs. Compared with needle injection, it induced distinct local tissue responses and immune-related transcriptional changes, suggesting that it may influence the cutaneous immune microenvironment. These findings represent distinctive features of needle-free vaccine delivery and provide preliminary evidence supporting its potential as an alternative vaccine administration approach.

## Data Availability

The original contributions presented in this study are included in the article. Further inquiries can be directed to the corresponding authors.

## Supplementary Materials

The following supporting information can be downloaded at: https://www.mdpi.com/article/10.3390/vetsci13090854/s1, Table S1. PCR primer sequences of each target gene; Table S2. Data for sequencing quality control. Raw reads: total number of reads in raw data; Clean reads: number of reads with adapters, low quality and quality score below Q20 removed; Total mapped reads: number of reads compared to the reference genome; Mapped rate (%): the percentage of clean reads mapped to the reference genome; ≥Q30 (%): the total number of bases with 99.9% or higher base identification accuracy in clean data; Figure S1. qRT-PCR validation and relative expression of some cytokine-related differential genes in the N, NI and NFI groups. (a) Relative expression of *CCL2 *gene in N, NI and NFI groups; (b) Relative expression of *CCRL2* gene in N, NI and NFI groups; (c) Relative expression of *CCL4 *gene in N, NI and NFI groups; (d) Relative expression of *CCL5* gene in N, NI and NFI groups; (e) Relative expression of *CXCL6* gene in N, NI and NFI groups; (f) Relative expression of *CCR7 *gene in N, NI and NFI groups; (g) Relative expression of *CCR9* gene in N, NI and NFI groups; (h) Relative expression of *CXCL10 *gene in N, NI and NFI groups; (i) Relative expression of *CXCL11 *gene in N, NI and NFI groups; (j) Relative expression of *ACKR3* gene in N, NI and NFI groups; (k) Relative expression of *ACKR4* gene in N, NI and NFI groups; N: blank control group; NI: one-day needle injection group; NFI: one-day needle-free injection group; ns: no statistical difference, * *P* < 0.05, ** *P* < 0.01; Figure S2. qRT-PCR validation and relative expression of some cytokine-related differential genes in the N, NI and NFI groups. (a) Relative expression of *XCL1 *gene in N, NI and NFI groups; (b) Relative expression of *INOS* gene in N, NI and NFI groups; (c) Relative expression of *IL10* gene in N, NI and NFI groups; (d) Relative expression of* ICAM1 *gene in N, NI and NFI groups; (e) Relative expression of *IL8* gene in N, NI and NFI groups; (f) Relative expression of *IL6* gene in N, NI and NFI groups; (g) Relative expression of *ICOS* gene in N, NI and NFI groups; (h) Relative expression of* PPBP *gene in N, NI and NFI groups; (i) Relative expression of* IL7* gene in N, NI and NFI groups; (j) Relative expression of *CCR1 *gene in N, NI and NFI groups; (k) Relative expression of* CCL19 *gene in N, NI and NFI groups; N: blank control group; NI: one-day needle injection group; NFI: one-day needle-free injection group; ns: no statistical difference, * *p* < 0.05, ** *p* < 0.01; Figure S3. qRT-PCR validation and relative expression of some cytokine-related differential genes in the N, NFI and NFS groups. (a) Relative expression of *CXCL10 *gene in N, NFI and NFS groups; (b) Relative expression of *XCL1* gene in N, NFI and NFS groups; (c) Relative expression of *PPBP* gene in N, NFI and NFS groups; (d) Relative expression of *INOS* gene in N, NFI and NFS groups; (e) Relative expression of* IL10 *gene in N, NFI and NFS groups; (f) Relative expression of *IL8* gene in N, NFI and NFS groups; (g) Relative expression of *CXCL11 *gene in N, NFI and NFS groups; (h) Relative expression of* CXCL14 *gene in N, NFI and NFS groups; (i) Relative expression of *IL6* gene in N, NFI and NFS groups; N: blank control group; NFI: one-day needle-free injection group; NFS: seven-day needle-free injection group; ns: no statistical difference, * *p* < 0.05, ** *p* < 0.01, *** *p* < 0.001, **** *p* < 0.0001; Figure S4. qRT-PCR validation and relative expression of some cytokine-related differential genes in the N, NFI and NFS groups. (a) Relative expression of *CXCL6* gene in N, NFI and NFS groups; (b) Relative expression of *CCRL2 *gene in N, NFI and NFS groups; (c) Relative expression of *CCR9 *gene in N, NFI and NFS groups; (d) Relative expression of *CCL2* gene in N, NFI and NFS groups; (e) Relative expression of *CCL5 *gene in N, NFI and NFS groups; (f) Relative expression of *CCL4* gene in N, NFI and NFS groups; (g) Relative expression of *CCR7 *gene in N, NFI and NFS groups; (h) Relative expression of *CCR5* gene in N, NFI and NFS groups; (i) Relative expression of *CCR1* gene in N, NFI and NFS groups; N: blank control group; NFI: one-day needle-free injection group; NFS: seven-day needle-free injection group; ns: no statistical difference, * *p* < 0.05, ** *p* < 0.01, *** *p* < 0.001, **** *p* < 0.0001.

## Abbreviations

The following abbreviations are used in this manuscript:

ACKR	Atypical Chemokine Receptor
BP	Biological Process
BSA	Bovine Serum Albumin
CC	Cellular Component
CCL	C-C Motif Chemokine Ligand
CCR	C-C Motif Chemokine Receptor
cDNA	complementary deoxyribonucleic acid
DAB	Diaminobenzidine
DEGs	Differentially Expressed Genes
ELISA	Enzyme-Linked Immunosorbent Assay
FDR	False Discovery Rate
FPKM	Fragments Per Kilobase of Transcript per Million Mapped Reads
*GAPDH*	Glyceraldehyde-3-Phosphate Dehydrogenase
GO	Gene Ontology
GSEA	Gene Set Enrichment Analysis
HRP	Horseradish Peroxidase
ICAM1	Intercellular Adhesion Molecule 1
IL	Interleukin
*INOS*	Inducible Nitric Oxide Synthase
KEGG	Kyoto Encyclopedia of Genes and Genomes
LC	Langerhans Cell
MF	Molecular Function
NES	Normalized Enrichment Score
NF-κB	Nuclear Factor kappa B
OD	Optical Density
PBS	Phosphate-Buffered Saline
PCV2	Porcine Circovirus Type 2
pNPP	p-Nitrophenyl Phosphate
qRT-PCR	Quantitative Real-Time Polymerase Chain Reaction
RNA	Ribonucleic Acid
RNA-seq	RNA sequencing
S/P ratio	Sample-to-Positive Ratio
SIRP-α	Signal Regulatory Protein Alpha
