# Supplementary material for: Enhanced Immunogenicity and Distinct Local Tissue Responses of Porcine Circovirus Type 2 Vaccination in Pigs Using a Needle-Free Jet Injection System
Source: Vet Sci. 2026 Aug 23;13(9):854. doi: 10.3390/vetsci13090854 (PMC13611694; doi:10.3390/vetsci13090854)
Supplement: Supplementary file 1 [file vetsci-13-00854-s001.zip › vetsci-4431887-supplementary.pdf]

Table S1: PCR primers sequences of each target gene.

Target Gene	Forward Primers (5′ to 3′)	Reverse Primers (5′ to 3′)
GAPDH	ATGGTGAAGGTCGGAGTGAA	CGTGGGTGGAATCATACTGG
CCRL2	TCTTCATCACGGTGTTCTTGGTTGG	GTTACACACTGCCAGGTTGAGGAG
IL6	AGACCCTGACGCAAAAGGGAAAG	CTCAGGTGCCCCAGCTACATTAT
IL10	AGAGTGCCTTTAGCAAGCTCC	TAGAGTCGTCATCCTGGAAGGT
IL7	CGCAGACCATGTTCCATGTTT	CAAAGTCTATCATCCTGTCCAAGTC
CXCL14	GCACTGCGAGGAGAAGATGGTTATC	TTCCAGGCGTTGTACCACTTGATG
CXCL10	CCACATGTTGAGATCATTGCCAC	CCACATGTTGAGATCATTGCCAC
CXCL11	TACAACTATTCAAGGCTTCCCC	GTCCTTTATGTGCTTTCAGGGT
CCL19	CTGGTTCGAGCCTTTCGCTA	GCGGTGGTGCTTGCTCTTAG
CCR7	CAGAAGAGCAGCAGCGAGCAG	GGATGATGACGAGGTAGCAGAAGC
IL8	TACGCATTCCACACCTTTCCA	GTTGTTGCTTCTCAGTTCTCTTCAA
CXCL6	TCCAAGGCAGAAGTGATAGCC	CCCACTGTCCAACATTTTCTGG
CCL5	TGGAGTTGAGCTAGGATGGAGGAAG	GCTGCTGTAAGTGGTGGAGTCTG
CCR9	CGACGCCTACACCATGTTCATCTC	GCGGAACCTCTCACCCACAAAC
ACKR3	AAGCACAGCAGCCGCAAGATC	GAGAAGATGTCCAGCAGCACCAC
CCL4	GACCATGAAGCTCTGCGTGACTG	GTGTATGTGAAGCAGCAGGAGGTG
CCL2	ACCAGCAGCAAGTGTCCTAAAGAAG	GGAGTCAGGCTTCAAGGCTTCG
XCL1	GCCTTCCTCGTGATCTGCTGTC	TGGAGCCCTCCTTGATGGTGTAG
ACKR4	CTGCCTGCCTTCTTCACAGTAGC	TCTGCCACTGCCAAATTCAGGATG
INOS	GCTGCCACCACGGAACCTAATG	AAAGAGTTGGAGAGGGAGGGAGATG
ICAM1	GCTCAGTGTCCTGTATGGACC	TGCCTGTAATCTCCCGCTTG
ICOS	GCTGGACCAACAGATGTGAC	GAGTCACGGGTTGAGACTGA
PPBP	TCCAGGTATTGCTGCCATTGTCAC	GCCAGAGACGGTATTCAAACAGAGG
CCR1	GGACCCATCAGCAGAACCACAAC	GCCTCCTCTACCTCCTACATCAACC

TableS2 Data to sequencing quality control

Sample	Raw Reads	Clean Reads	Total Mapped Reads	Mapped rate(%)	≥Q30(%)
N1	38179202	35957436	34527878	96.02	92.05
N2	44781780	42269444	40457025	95.71	91.83
N3	44516368	41789428	40308107	96.46	93.41
NI1	40105520	38131582	36278033	95.14	92.39
NI2	51578094	49022064	47168936	96.22	94.18
NI3	45737284	43476246	41435591	95.31	92.56
NFI1	46949494	44840492	42679684	95.18	92.47
NFI2	46633642	44470876	42132784	94.74	93.63
NFI3	42257486	40286430	38596951	95.81	93.34
NFS1	45972202	43843934	42028499	95.86	93.33
NFS2	43693678	41674692	39910752	95.77	93.23

NFS3	43606778	41621320	39858720	95.77	93.19

Note: Raw reads: total number of reads in Raw data; Clean reads: number of reads with splice, low quality and quality score below Q20 removed; Total Mapped Reads: number of reads compared to the reference genome; Mapped rate (**%)** of reads compared to the reference genome as a percentage of Clean reads reads as a percentage of Clean reads; ≥ Q30(%): the total number of bases with 99.9% or higher base identification accuracy in Clean data.

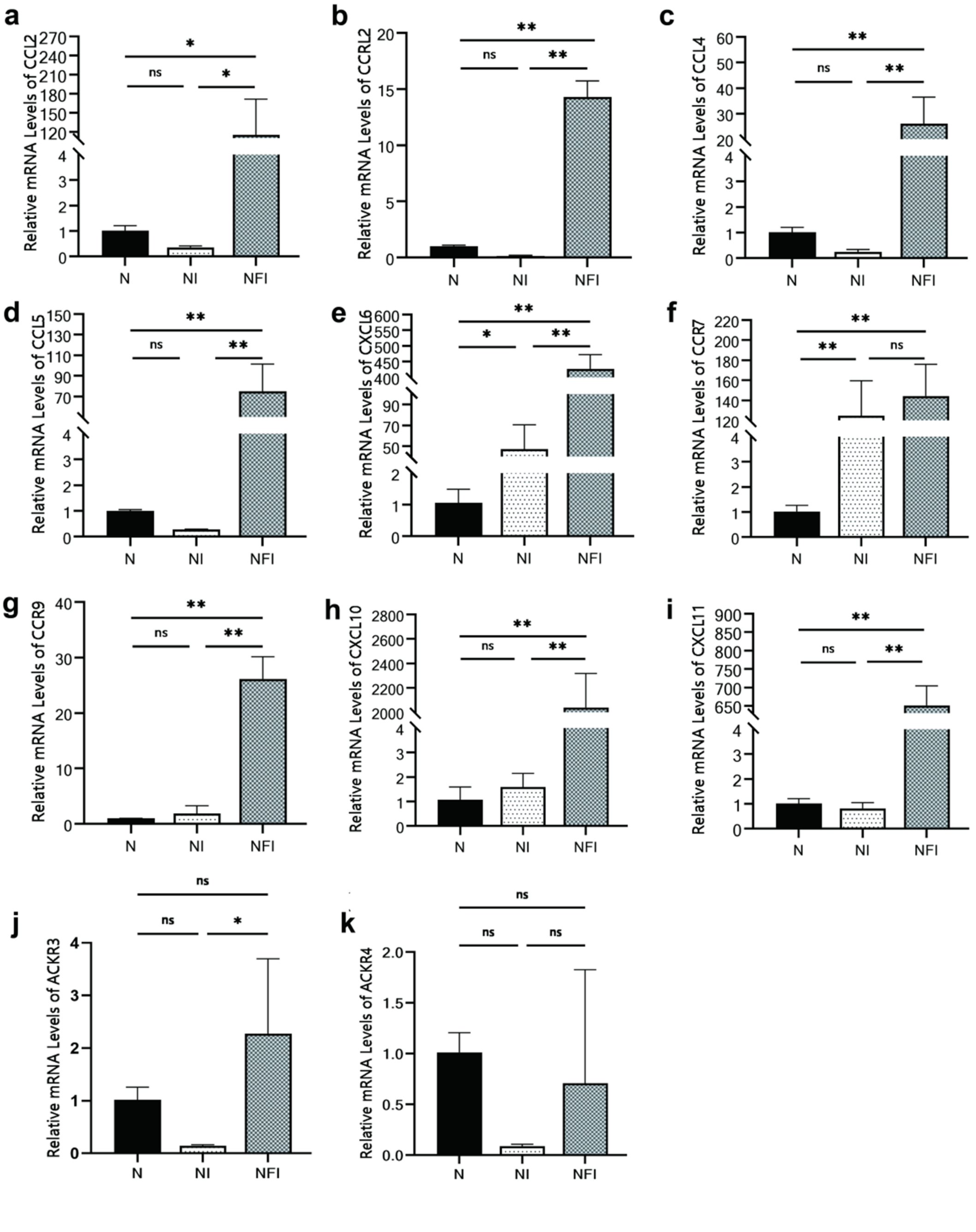

Figure S1. qRT-PCR validation and relative expression of some cytokine-related differential genes in the N, NI and NFI groups. (a) Relative expression of *CCL2* gene in N, NI and NFI groups; (b) Relative expression of *CCRL2* gene in N, NI and NFI groups; (c) Relative expression of *CCL4* gene in N, NI and NFI groups; (d) Relative expression of *CCL5* gene in N, NI and NFI groups; (e) Relative expression of

CXCL6 gene in N, NI and NFI groups; (f) relative expression of *CCR7* gene in N, NI and NFI groups; (g) relative expression of *CCR9* gene in N, NI and NFI groups; (h) relative expression of *CXCL10* gene in N, NI and NFI groups; (i) relative expression of *CXCL11* gene in N, NI and NFI groups; (j) relative expression of *ACKR3* gene in N, NI and NFI groups; (k) relative expression of *ACKR4* gene in N, NI and NFI groups; N : blank control group; NI: one day intradermal injection group; NFI: one day needle-free injection group; ns: no statistical difference, *$P < 0.05$, ** $P < 0.01$.

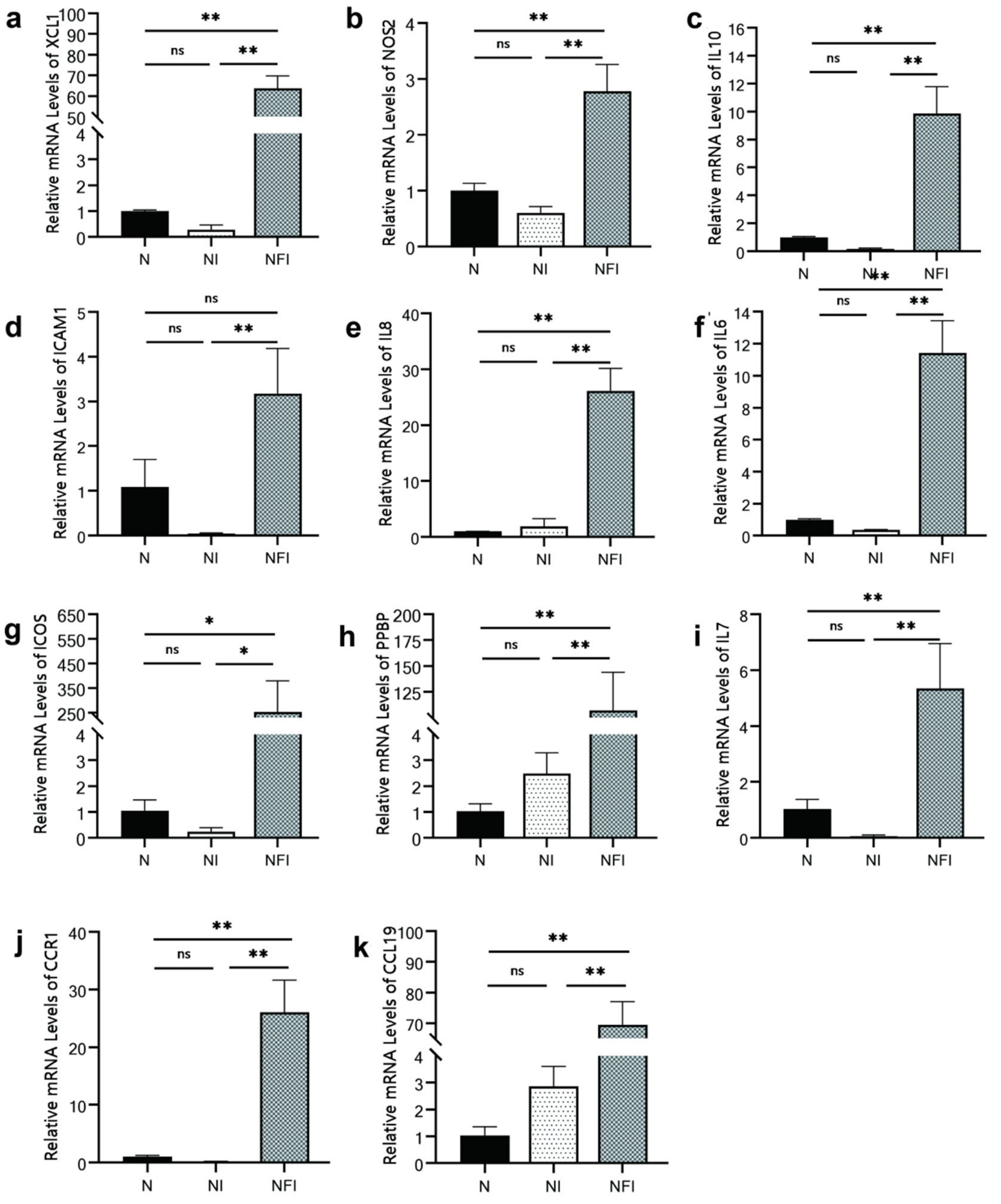

Figure S2. qRT-PCR validation and relative expression of some cytokine-related differential genes in the N, NI and NFI groups. (a) Relative expression of *XCL1* gene in N, NI and NFI groups; (b) Relative expression of *INOS* gene in N, NI and NFI groups; (c) Relative expression of *IL10* gene in N, NI and NFI groups; (d) Relative expression of *ICAM1* gene in N, NI and NFI groups; (e) Relative expression of *IL8* gene in N, NI and NFI groups; (f) relative expression of *IL6* gene in N, NI and NFI groups; (g) relative expression of *ICOS* gene in N, NI and NFI groups; (h) relative expression of *PPBP* gene in N, NI and NFI groups; (i) relative expression of *IL7* gene in N, NI and NFI groups; (j) relative expression of *CCR1* gene

in N, NI and NFI groups; (k) relative expression of *CCL19* gene in N, NI and NFI groups; N : blank control group; NI: one day intradermal injection group; NFI: one day needle-free injection group; ns: no statistical difference, *$P < 0.05$, ** $P < 0.01$.

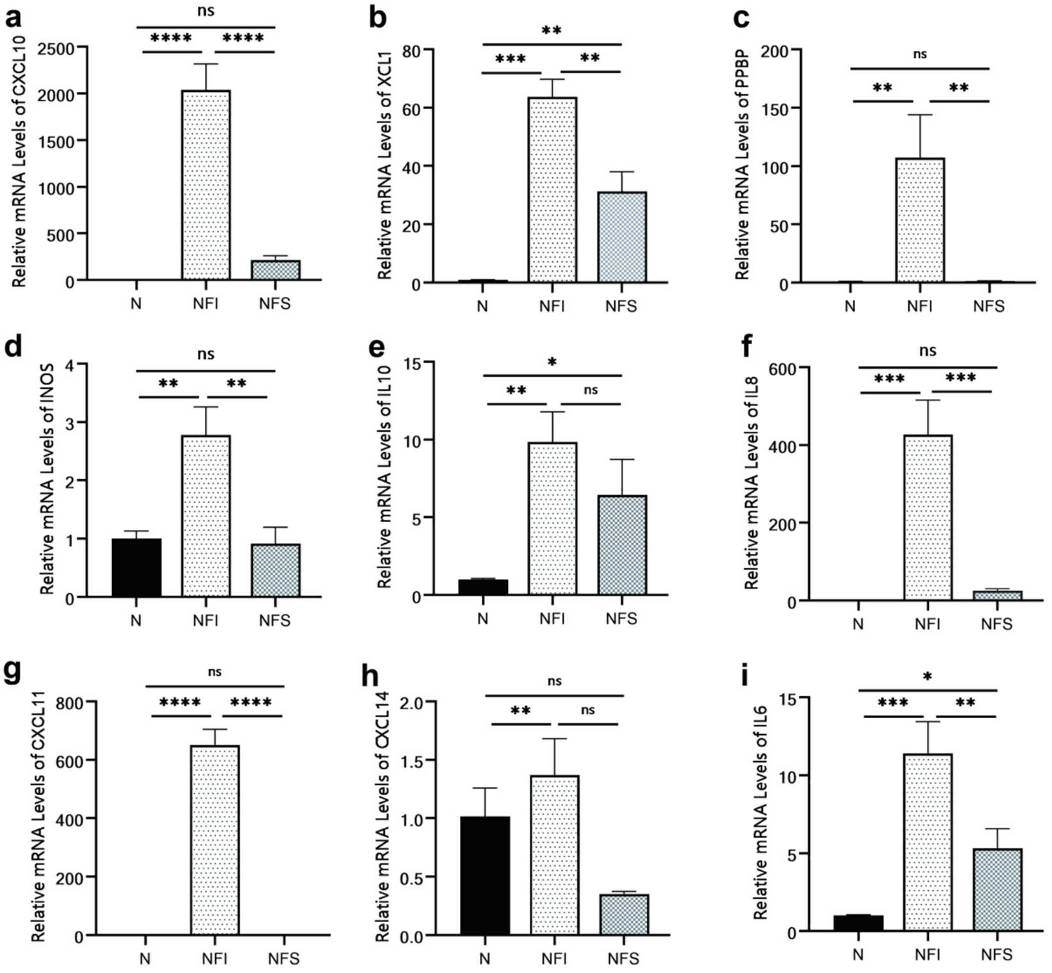

Figure S3. qRT-PCR validation and relative expression of some cytokine-related differential genes in the N, NFI and NFS groups. (a) Relative expression of *CXCL10* gene in N, NFI and NFS groups; (b) Relative expression of *XCL1* gene in N, NFI and NFS groups; (c) Relative expression of *PPBP* gene in N, NFI and NFS groups; (d) Relative expression of *INOS* gene in N, NFI and NFS groups; (e) Relative expression of *IL10* gene in N, NFI and NFS groups; (f) Relative expression of *IL8* gene in N, NFI and NFS groups; (g) Relative expression of *CXCL11* gene in N, NFI and NFS groups; (h) Relative expression of *CXCL14* gene in N, NFI and NFS groups; (i) Relative *IL6* gene in N, NFI and NFS groups expression; N: blank control group; NFI: one-day needle-free injection group; NFS: seven-day needle-free injection group; ns: no statistical difference, *$P < 0.05$, ** $P < 0.01$, ***$P < 0.001$,****$P < 0.0001$.

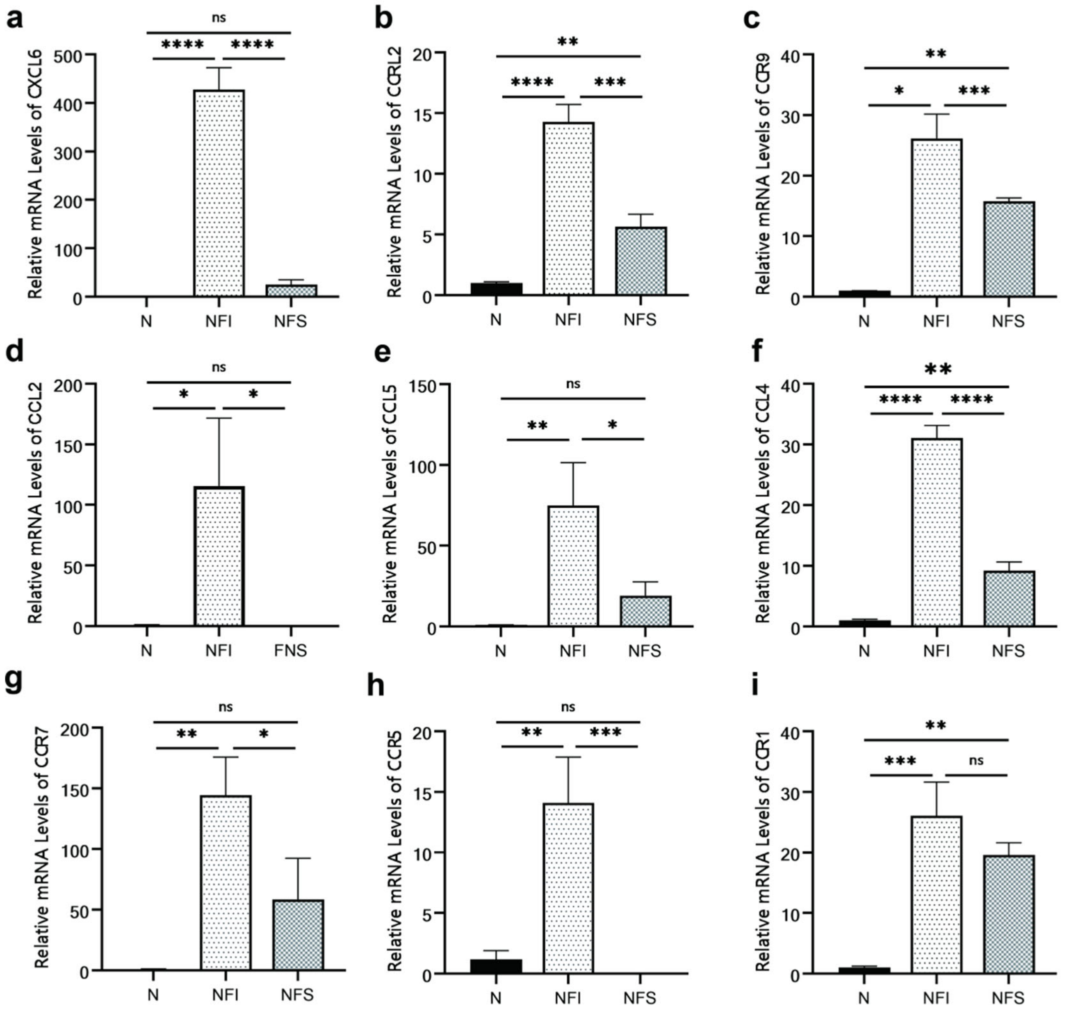

Figure S4. qRT-PCR validation and relative expression of some cytokine-related differential genes in the N, NFI and NFS groups. (a) Relative expression of *CXCL6* gene in N, NFI and NFS groups; (b) Relative expression of *CCRL2* gene in N, NFI and NFS groups; (c) Relative expression of *CCR9* gene in N, NFI and NFS groups; (d) Relative expression of *CCL2* gene in N, NFI and NFS groups; (e) Relative expression of *CCL5* gene in N, NFI and NFS groups; (f) relative expression of the *CCL4* gene in the N, NFI and NFS groups; (g) relative expression of the *CCR7* gene in the N, NFI and NFS groups; (h) relative expression of the *CCR5* gene in the N, NFI and NFS groups; (i) relative expression of the *CCR1* gene in the N, NFI and NFS groups; N: blank control group; NFI: one-day needle-free injection group; NFS:seven-day needle-free injection group; ns: no statistical difference, *$P < 0.05$, ** $P < 0.01$, ***$P < 0.001$, ****$P < 0.0001$.
